# Healing Activity of Propolis of Stingless Bee (*Scaptotrigona aff. postica*), Reared in Monoculture of Açaí (*Euterpe oleracea*), in Induced Wounds in Rats

**DOI:** 10.3390/molecules29194742

**Published:** 2024-10-07

**Authors:** Sara R. L. Ferreira, Suzanne A. Teixeira, Gabriella O. Lima, Jhennifer N. R. S. de Castro, Luís E. O. Teixeira, Carlos A. R. Barros, Daniel S. Pereira, Moisés Hamoy, Veronica R. L. O. Bahia, Nilton A. Muto

**Affiliations:** 1 Center of the Valorization of Amazonian Bioactive Compounds—CVACBA, Federal University of Pará, Belém 66075-750, Pará, Brazil; sara.ferreira@icb.ufpa.br (S.R.L.F.); suzanneteixeirac2@gmail.com (S.A.T.); luis.teixeira@icb.ufpa.br (L.E.O.T.); carlos.barros@icb.ufpa.br (C.A.R.B.); 2 Department of Histology, Biological Sciences Institute, Federal University of Pará, Belém 66075-110, Pará, Brazil; gabbylimaoli13@gmail.com (G.O.L.); jhennifer.silva@ics.ufpa.br (J.N.R.S.d.C.); vrlo@ufpa.br (V.R.L.O.B.); 3 Embrapa Eastern Amazon, Belém 66095-903, Pará, Brazil; daniel.pereira@embrapa.br; 4 Laboratory of Pharmacology and Toxicology of Natural Products, Biological Sciences Institute, Federal University of Pará, Belém 66075-110, Pará, Brazil; hamoyufpa@gmail.com

**Keywords:** healing activity, propolis, stingless bee, *Euterpe oleracea*

## Abstract

Wound healing is a complex and coordinated process involving interactions between cells and various messenger systems. This study conducted in vivo tests to determine the healing effect of propolis (PR)-based cream derived from the Amazon stingless bee, *Scaptotrigona aff. postica,* reared in açaí (*Euterpe oleracea*) monoculture, on induced wounds in rats. Data were obtained by monitoring injuries on 14 Wistar rats, divided into three groups (G1, G2 and G3), each receiving specific treatments: propolis-based cream (PR), collagenase (PC) and neutral cream (NC). Over the seven days of treatment, the lesions were measured using photographic records and ImageJ software to evaluate the healing effectiveness of the test cream. ImageJ software version 1.53g was used to compare the wound diameters for each treatment. After seven days, histopathological analyses of the induced lesions were performed. It was observed that collagenase (PC) and the test cream (PR) did not differ significantly in terms of wound diameter reduction. However, the propolis-based cream directly influenced the lesion maturation process and exhibited a milder inflammatory response compared to the positive control (PC). This effect is possibly associated with antimicrobial and anti-inflammatory compounds identified by GC/MS analysis in the propolis. Notably, this is the first report describing propolis of *Scaptotrigona aff. postica* obtained from açaí monocultures with strong healing potential, highlighting the identification of a high concentration of phenolic compounds that aid directly in wound repair.

## 1. Introduction

Propolis has been utilized by humans since antiquity as one of the most versatile bioproducts. It is naturally produced by bees through a mixture of resinous substances derived from plant resins, combined with beeswax, pollen, and salivary secretions [1]. The composition of active principles that promote unique and health-beneficial properties is composed of over 800 different components, with flavonoids, esters, phenolic acids, and phenolic aldehydes being particularly significant due to their notable antioxidant and antimicrobial activities. The variation in the percentage of these constituents in propolis depends on the collection period and geographical origin. These properties are commonly applied in pharmacology, medicine, cosmetology, and the food industry, among others [2].

Recent scientific studies have identified its therapeutic potential through anti-inflammatory, antimicrobial, and antineoplastic activities [3]. Additionally, the antioxidant compounds are the main modulators of the immune system and assist in the absorption of vitamins that are crucial in the healing process [4].

Native bees (*Scaptotrigona aff. postica*) are located in Brazil, specifically in the Amazon region, and are characterized by a sting atrophy during their development; hence, they are called stingless bees. They are noted for their high pollination of tropical forests and production of honey, wax, and propolis. The propolis produced by these bees has been studied for its biological activities. However, it is important to note that variables closely related to the biodiversity of the plants visited by stingless bees can alter the chemical composition of the produced propolis, due to the diversity of collected plant resins and pollen. This diversity can influence the synthesis of propolis and enhance its therapeutic potential [5]. According to Lavina et al. (2019), certain chemical compounds such as flavonoids and terpenoids are found in high concentrations in the propolis of stingless bees, thereby enhancing its wound-healing and antimicrobial activities [6].

The açaí palm (*Euterpe oleracea*) is highly valued for its high concentrations of phenolic compounds and anthocyanins, which have significant antioxidant capacity. For instance, a study by Pattanayak et al. (2008) demonstrated that the topical application of antioxidant compounds resulted in significant improvement in wound healing, providing tissue protection against oxidative damage [7].

The constant availability of a major floral source, as in monoculture environments, results in a more uniform chemical composition of propolis, rich in specific bioactive compounds. Thus, the monoculture of açaí creates a specific environment that directly influences the characteristics and composition of propolis produced by stingless bees (*Scaptotrigona aff. postica*).

The foraging radius of *Scaptotrigona* bees is generally reported in the scientific literature as ranging from 600 to 900 m. However, research conducted in açaí-growing areas, including studies conducted with Muto et al. (2020) and with researcher Campbell et al. (2023), demonstrate that the foraging radius in these specific regions is significantly smaller, ranging from 180 to 300 m. It is important to note that, based on current evidence, it is not possible to definitively determine the origin of the collected propolis; it is only necessary to speculate about its possible origin based on the range of the bees and the extent of the areas explored [8,9].

Although previous studies have explored the therapeutic properties of propolis, this study stands out by investigating the healing effect of a cream based on propolis produced by stingless bees (*Scaptotrigona aff*. *postica*) raised in açaí monoculture at a distance of 0 to 50 m, an unprecedented approach that explores the direct influence of local flora on the therapeutic efficacy of propolis, specifically in wounds induced in rats.

## 2. Results

### 2.1. Total Phenol Content Results

According to the literature, to determine the content of phenolic substances present in propolis, it is necessary to use the Folin–Ciocalteu reagent as a reference substance [10]. In this sense, using this approach in this study, Table 1 represents the spectrophotometric assays carried out with the hydroethanolic extract of propolis, expressed in gallic acid. In this way, it is possible to determine the number of total phenols in the sample, obtaining a value for the highest absorbance with 5.04% with a concentration of 5471.22 mg/L of gallic acid. These results are in accordance with the minimum requirements postulated by the Brazilian Ministry of Agriculture (2001) [11]. These are satisfactory results since in some studies carried out, the phenolic content was lower than that found in the present work, as in the study by [12], in which the authors performed spectrophotometric analyses of propolis from different regions of Argentina, studying the best colorimetric method, obtaining a content of 1.23% of total phenolic compounds. It is evident that these results bring satisfactory perspectives for propolis of Amazonian origin since several authors have already demonstrated that Brazilian propolis has a high percentage of phenolics.

### 2.2. Flavonoid Content Results

To avoid interference from other classes of compounds that may be present in the sample, especially phenolic acids, we adopted a quantification methodology based on the aluminum cation property, responsible for the formation of stable complexes with flavonoids, leading to a shift towards greater enhancement of their absorbances [13]. The value found for the total average of absorbances was 0.191. This value is close to that found in the study by Funari et al. (2006) for samples of Brazilian crude propolis [14]. It is necessary to mention that the cited study is based on the reaction of aluminum chloride and flavonoids.

Thus, the results obtained in the analysis of phenolics present in propolis explain why some authors attribute these compounds to the therapeutic effects present in propolis. Among the classes of compounds, flavonoids may be primarily responsible for the healing property found in propolis so that there is a rapid recovery of injured tissue, through biochemical and morphological phenomena [15, 16]. In addition, the results indicate excellent samples, since, as previously mentioned, the bioactive compounds are responsible for the biological healing activity of the product.

### 2.3. Gravimetric Results

The results of the analysis of loss due to desiccation at 105 °C in an oven, as well as humidity, are within the limit established by the Brazilian Ministry of Agriculture (2001) [11], where humidity and ash content cannot exceed 8% and 5%, respectively. These percentages certify the quality of propolis, since its moisture obtained an average value of 7.8%, demonstrating that there was no great influence of rainfall on the values obtained, as can be seen in Table 2.

Ash analysis shows the degree of non-volatilizable residual substances present in the sample [15]. This is one of the most relevant analyses in the study, as it may indicate a possible adulteration in the product, such as impurities, especially earth, or other residues arising at the time of extraction. The values obtained are satisfactory, as they are in accordance with the limit established by the responsible agency. The averages indicate a value of 3.19% in relation to the mass of crude propolis (*m*/*m*) as can be analyzed in Table 2.

### 2.4. GC-MS Results

The GC-MS analysis revealed high levels of major compounds, all belonging to the class of triterpenes or triterpenoids, derived from six isoprene units (C_5_H_8_), with structures based on 30 carbon atoms. These compounds include both pentacyclic triterpenes, characterized by five carbon rings, and those with important functional modifications such as hydroxyl or ketone groups and esterification by acetates.

The most abundant compound, lup-20(29)-en-3-one (32.64%), is a triterpene with a double bond between carbons C-20 and C-29 and a ketone group at position C-3, belonging to the lupane class, derived from a basic skeleton of 30 carbon atoms; lupeol (17.21%) is another pentacyclic triterpene, with a hydroxyl group (-OH) attached to one of the carbon atoms in the ring. Its molecular formula is C_30_H_50_O, and like other triterpenoids, it is derived from squalene, an important precursor in the biosynthesis of steroids; 4,4,6a,6b,8a,11,11,14b-octamethyl-1,4,4a (13.16%) belongs to the class of highly branched polycyclic hydrocarbons, possibly derived from complex structures like norbornanes or steroids; lup-20(29)-en-3-ol, acetate, (3β)- (9.93%) is also part of the lupane class, being a triterpene esterified with an acetate group at position C-3; β-amyrin (7.09%) is a pentacyclic triterpene from the oleanane subclass, a structure derived from the polymerization of six isoprene units, typical of many bioactive triterpenes; olean-12-en-ol (2.54%) is another example of a pentacyclic triterpene, belonging to the oleanane subclass, originating from squalene, the common precursor of many steroids and other biologically active molecules; acetate, (3β)- (2.06%), refers to a compound esterified by acetate, with a 3β-hydroxyl configuration, commonly found in esterification reactions with alcohols and steroids.

**Figure 1:**
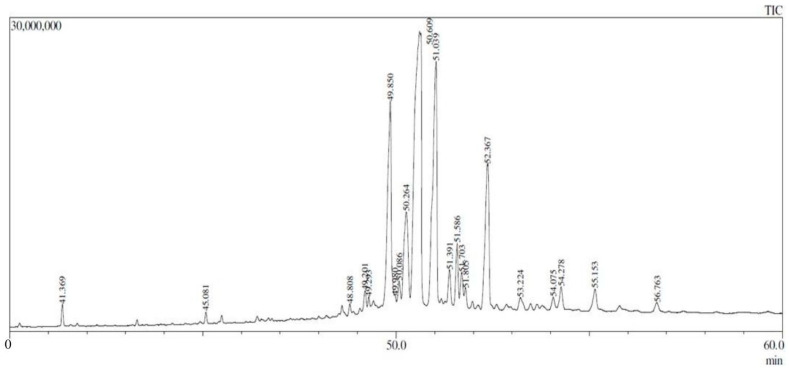
GC-MS analysis of compounds present in ethanolic extract of *Scaptotrigonna aff. postica* propolis reared in açaí monoculture. Major compounds at retention time (RT) of 49.850, 50.609, 51.039, and 52.367 in min.

The chemical characterization, identified by the molecular fragmentation pattern and compared to mass spectral data, revealed the presence of 27 compounds. These compounds are notable for their biological functions and relevance in the biosynthesis of steroids and other biologically active terpenoids (Figure 1 and Table 3).

### 2.5. Macroscopic Results

Throughout the entire experimental period of this study, the animals recovered well in the postoperative period, showing good healing progression. The ImageJ software was utilized to measure the wound diameters and the statistical analysis was conducted using Biostat 5.0 and GraphPad Prism 8.

**Figure 2:**
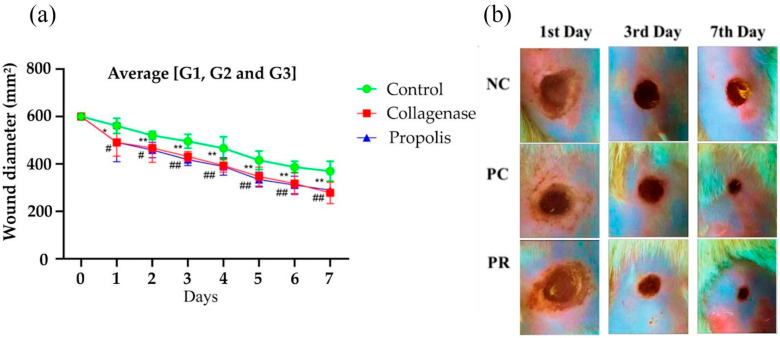
(**a**) Morphometric analysis of the wound area demonstrating macroscopic evolution of the wounds on the 1st, 3rd and 7th day of the in vivo test, starting from the dorsal region to the caudal region with negative control (NC), collagenase (PC) and propolis-based cream (PR). (**b**) Mean wound diameter in groups of rats G1, G2 and G3 with temporal comparison from 0 to 7 days: control (NC), collagenase (PC) and propolis-based cream (PR). Statistically significant difference between the PR and NC (*); *p* < 0.05 (*); *p* < 0.01 (**); (#) statistical difference in the participation of collagenase in relation to the control group: *p* < 0.05 (#); *p* < 0.01 (##).

The wounds treated with propolis-based cream (PR) exhibited satisfactory results over the 7-day period, demonstrating significant differences compared to the negative control (NC). On the first day, a significant difference (*p* < 0.05) was observed between propolis (PR) and negative control (NC). In the subsequent days, the propolis-based cream (PR) consistently exhibited smaller wound diameters compared to negative control (NC), demonstrating that it had a higher healing efficiency (Figure 2) with a statistically significant difference (*p* < 0.01). The propolis-based cream (PR) and collagenase (PC) showed statistically identical performance, with their wound diameters overlapping, indicating similar macroscopic effectiveness (Figure 2).

### 2.6. Histopathological Results

**Figure 3:**
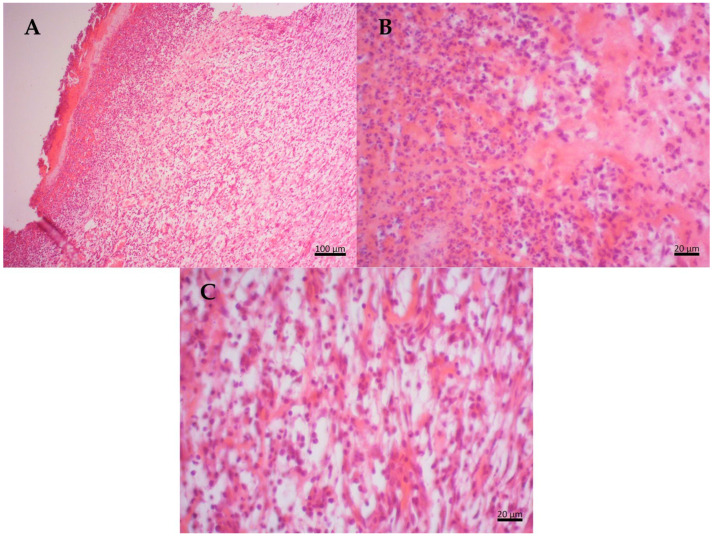
Histological section of Wistar rat wounds at day 7 after treatment. Negative control (NC)—(**A**) an intense inflammatory response with intense leukocyte activity is observed on the tegument surface; (**B**) the presence of inflammatory exudate; (**C**) in the deepest region of the dermis, the formation of granulation tissue with the presence of several blood vessels and fine collagen fibers is observed and the presence of inflammatory cells and fibroblasts.

Histopathological analysis was performed on the 7th day of the in vivo experiment using hematoxylin/eosin staining. As shown in Figure 3, the negative control group exhibited a marked inflammatory response with intense leukocyte activity, the presence of inflammatory exudate, and the formation of loose connective tissue with noticeable blood capillaries. Additionally, there were possible retractions in the inflammation within the lesion. The presence of granulation tissue with a pink and granular appearance was also observed.

**Figure 4:**
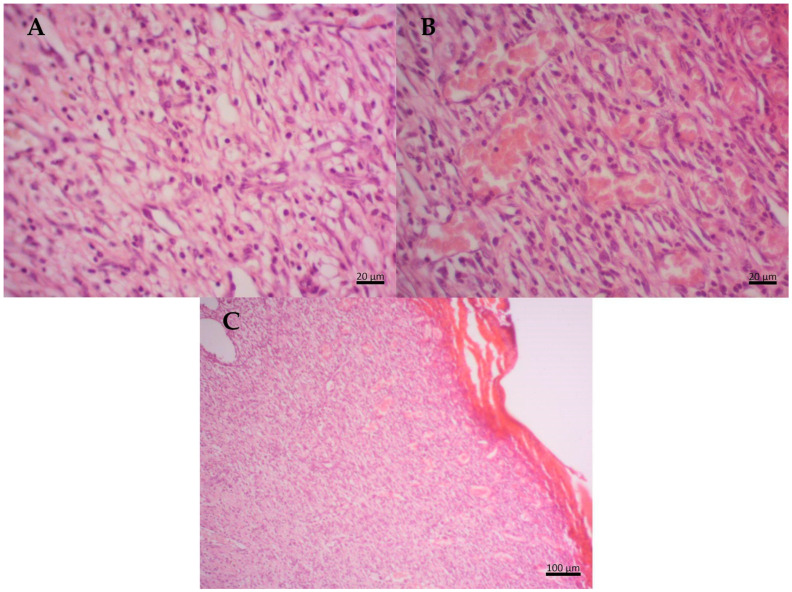
Histological section of Wistar rat wounds at day 7 after treatment with collagenase (PC)—(**A**) intense proliferation of fibroblasts with several type III collagen fibers; (**B**) the presence of blood vessels; (**C**) observe the granulation tissue on the surface of the dermis.

In the positive control (PC), the samples showed a moderate inflammatory response. There is an intense proliferation of fibroblasts with the presence of several type III collagen fibers and reticular fibers, characteristics of dense connective tissue. Additionally, a large number of blood capillaries are noticeable on the surface of the wound, indicating intense vascularization. Furthermore, there is evidence of a re-epithelialization process at the edge of the lesion, suggesting ongoing wound healing (Figure 4).

**Figure 5:**
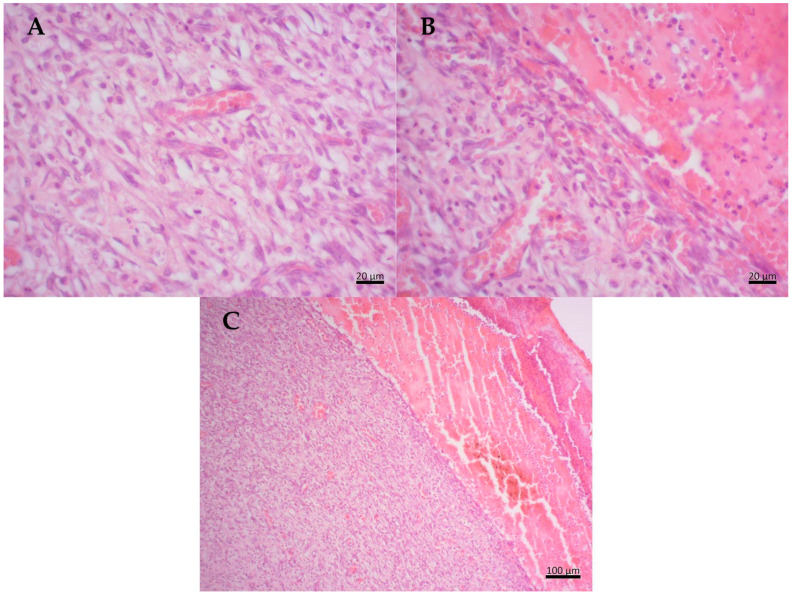
Histological section of Wistar rat wounds at 7 days after treatment with propolis-based cream (PR). (**A**) Presence of type I and type III collagen fibers; (**B**) presence of fibroblasts; (**C**) highly organized granulation tissue with abundant blood vessels and more densely organized keratinized stratum corneum.

In the propolis-based treatment, the groups showed a moderate inflammatory response, a little less intense than that observed in the positive control, suggesting the presence of a healing agent in the propolis-based cream that mitigates autoimmune response. There is notable proliferation of fibroblasts from the superficial region to the injury area, with abundant reticular and type I and III collagen fibers. Moderate proliferation of blood capillaries is observed, along with re-epithelialization at the wound edges. Additionally, there is a well-organized basement membrane, observed particularly in individuals treated with propolis-based cream (Figure 5).

## 3. Discussion

The study is pioneering in utilizing Amazonian propolis extract as an active ingredient in a biopharmaceutical cream, being the first to prove the therapeutic properties of propolis collected in a specific area, located between 0 and 50 m away, containing açaí nectar (*Euterpe oleracea*). This unique propolis is produced by the stingless bee species native to the Amazon, *Scaptotrigona aff. postica,* using Amazonian materials and floral nectars from *E. oleracea*. Existing research has primarily focused on propolis from other regions of Brazil, highlighting the variability in propolis composition influenced by factors such as geographical location, edaphoclimatic conditions, collection season, and bee species.

This study aimed to evaluate the efficacy of a propolis-based cream in healing induced wounds in Wistar rats, focusing on constituent compounds and observing macroscopic and microscopic changes over a seven-day in vivo test period. Comparison with collagenase, a well-established pharmaceutical agent, and a control cream devoid of active ingredients demonstrated significant healing benefits in macroscopic evaluation and histopathological analysis. Importantly, the propolis-based cream showed statistically similar efficacy to collagenase.

The primary objective of employing therapeutic protocols for injuries is to reduce healing time and optimize the quality of healed tissue to prevent future complications such as contamination or infection. Biopharmaceuticals such as propolis-based creams offer advantages such as minimal side effects, low chemical residues, and negligible preservatives. Propolis, known for its anti-inflammatory and antimicrobial properties, promotes increased vascularity and granulation tissue synthesis, as observed in this study. This therapeutic efficacy may be attributed to aromatic compounds, with phenolic compounds identified at concentrations exceeding 5.04% (*m*/*m*) in the propolis sample. Total phenolic content was determined using an oxi-reduction reaction method with the Folin–Ciocalteu reagent, though results may underestimate actual concentrations due to variability in analytical methods.

Quantification of total flavonoids showed approximately 1.91%, expressed as quercetin dihydrate equivalents, using spectrophotometric methods [10]. This value contrasts with higher concentrations observed using HPLC techniques in similar studies, indicating potential underestimation. Flavonoids are pivotal for skin re-epithelialization, influencing healing processes [15,23].

Gas Chromatography coupled with mass spectrometry (GC-MS) identified bioactive compounds in the propolis-based cream, including lup-20(29)-en-3-one, which constitutes 32.64% of total compounds. Known for hepatoprotective properties [18], lup-20(29)-en-3-one promotes antioxidant activity and cellular regeneration [21]. Other compounds such as lupeol, β-amyrin, and olean-12-en-ol acetate, identified with antioxidant, antitumor, and anti-inflammatory activities, contribute synergistically to wound healing [18,19,23].

The study by Alburqueque Junior et al. (2009) focused on assessing the wound-healing properties of propolis-based films using an experimental model with Wistar rats. Their findings confirmed propolis’ ability to promote wound healing by modulating collagen deposition and controlling inflammation progression [24]. This modulation has a key role in maintaining a balance between necessary inflammatory responses and excessive inflammation, which can impede the healing process.

Additionally, a study by Arvouet-Grand et al. (1994) [25] explored propolis’ therapeutic potential and its bioactive compounds in enhancing wound healing. The study emphasized honeybee propolis’ anti-inflammatory properties and its role in stimulating cellular regenerative processes, thus reinforcing the findings of our study. These validations across multiple studies underscore the potential of propolis-based creams derived from *Scaptotrigona aff. postica* in combating inflammation and promoting cellular regeneration, suggesting promising applications in developing treatments for human skin wounds.

The gravimetric results validate the quality of the collected propolis extract, aligning with limits set by the Brazilian Ministry of Agriculture (2001) [11]. The ash content averaged 3.155% (±0.098), within the permissible limit of 5%, while loss by desiccation averaged 3.155% (±0.002), below the maximum limit of 8%. These data confirm that the extract used is free from interferences that could compromise the study’s results.

Figure 2 illustrates that over the 7-day period, both propolis (PR) and collagenase-based (PC) creams demonstrated accelerated wound healing compared to the control group (NC). The effectiveness of the propolis-based cream was evaluated against collagenase (PC) and a neutral cream (NC), showing statistically significant differences compared to the NC group. By the 3rd day, PR exhibited a greater reduction in wound diameter compared to other groups (*p* < 0.01), measuring approximately 430 mm^2^ compared to above 580 mm^2^ for the others. Significant reductions in wound diameter (*p* < 0.01) were observed on the 3rd, 5th, and 7th days for both PR and PC compared to NC, highlighting superior healing outcomes.

Figure 2b shows the average group data, indicating a significant difference for the propolis-based cream compared to NC from the 3rd day onwards (*p* < 0.05), and significant differences compared to collagenase on the 1st, 3rd, 5th, and 7th days. Figure 5 illustrates a moderate inflammatory response on the 7th day of treatment, attributed to active compounds in propolis that aid in fibroblast synthesis, crucial for collagen fiber production. This process is evident through the formation of dense connective tissue enriched with reticular and type I collagen fibers, characteristic of granulation tissue formation and fibroplasia. Enhanced fibroblastic proliferation and vascularization contribute to accelerated wound healing.

Considering the stages of healing, the maturation phase exhibits a milder inflammatory response compared to the positive control. While collagenase acts by decomposing and synthesizing collagen to promote tissue regeneration and wound re-epithelialization, propolis’ effectiveness may stem from similar principles alongside its demonstrated antimicrobial and anti-inflammatory properties. Studies confirming propolis’ antimicrobial efficacy against Gram-negative bacteria further support its role in infection control during wound healing [26].

The healing effects of propolis are associated with its antimicrobial and anti-inflammatory properties, warranting future in vitro studies to substantiate these properties in Amazonian propolis. Overall, our study demonstrates macroscopic and microscopic equivalence in wound healing between propolis (PR) and collagenase (PC), both showing milder inflammatory responses than the positive control. Microscopic analysis of individuals treated with propolis-based cream revealed the presence of a potential basement membrane near the wound edge, indicative of accelerated re-epithelialization. Specific analyses are necessary to confirm the membrane’s constituents.

Thus, this study not only confirms the efficacy of Amazonian propolis but also opens new possibilities for its application in bioproducts aimed at wound healing. Extended evaluation beyond 7 days, as conducted in studies such as Krupp et al. (2019), would likely reveal a more intense healing phase and complete wound closure, providing further insights into propolis’ therapeutic potential [27].

## 4. Materials and Methods

### 4.1. Material Collection

Propolis samples were collected from beehives of straw bees (*Scaptotrigona aff. postica*) 12 months after installation in the center of an irrigated açaí cultivation area, with a cultivation radius greater than 600 m, in the municipality of Tomé Açú-PA, Brazil (2°33′20.2″ S 48°19′46.0″ W).

### 4.2. Preparation of Physicochemical Analysis

An extraction process was conducted by maceration for 10 days with ethanol/water (70% *v*/*v*) (1:10 *w*/*v* propolis ratio) to obtain hydroethanolic propolis extract. The extract was subsequently filtered and concentrated in a rotary evaporator at 50 °C at 70 rpm.

### 4.3. Physicochemical Analysis

The physicochemical analysis was conducted in accordance with the Brazilian Pharmacopoeia (2010) [28].

#### 4.3.1. Quantification of Total Polyphenols

The total polyphenol content was determined by the adapted Folin–Ciocalteu method [29] and expressed as milligrams of gallic acid equivalents per gram of dried extract (mg GAE/g DE).

#### 4.3.2. Quantification of Total Flavonoids

Flavonoids were quantified using quercetin dihydrate as a reference substance [30]. A standard curve was prepared from the reference substance, and a 2.5% aluminum chloride solution was used. After mixing 1 mL of this solution with 8.8 mL of propolis, the mixture was adjusted to 25 mL with ethanol.

#### 4.3.3. Determination of Ash Content

The ash content was determined by placing 1 g of pulverized propolis samples in a porcelain crucible with a known weight. The crucible was heated on a heating plate at 350 °C until no more smoke was produced. Subsequently, the crucible was transferred to a muffle furnace at 600 °C until the ashes turned completely white. The analysis was performed in triplicate, and the ash content was calculated as a percentage ratio of the ash mass to the initial mass of propolis.

#### 4.3.4. Loss on Drying at 105 °C

Raw and pulverized propolis samples (1 g) were placed in a porcelain crucible with a known weight and heated in an oven at 105 °C for 3 h. After cooling, the material was weighed. The analysis was performed in triplicate, and the loss on drying was calculated as a percentage ratio of the mass of the volatilized material to the initial mass of propolis.

### 4.4. Preparation of Propolis Extract for GC-MS

Propolis was pretreated at −80 °C for maceration. Nonpolar compounds were extracted by mixing 10 g of macerated propolis with methanol, followed by the addition of 100 mL of MilliQ water and 50 mL of dichloromethane. The mixture was stirred and left to settle for 20 min. The denser phase was dried in a chemical fume hood and analyzed by GC-MS [31].

#### Gas Chromatography

GC-MS analysis of the propolis dichloromethane extract from *Scaptotrigona aff. postica* was performed using a Shimadzu GCMS-QP2010 Plus equipped with a ZB-5HTS Inferno™ column (30.0 m length, 0.25 mm diameter, 0.25 μm thickness). Experimental parameters included a column oven temperature of 60 °C, helium carrier gas flow rate of 1.8 mL/min, injection mode splint ratio of 5.0 and an ion-source temperature of 280 °C. Mass spectrometry was conducted in full-scan mode with a scan range of 37 to 660 m/z. Volatile Organic Compounds (VOCs) were identified by matching their MS fragments with those in the Wiley Registry/NIST Mass Spectral Library.

### 4.5. Preparation of Propolis-Based Cream

The 10% *w*/*v* propolis-based cream was formulated using the phase inversion technique, as outlined by Golçalves (2000) [32], to ensure the formation of a stable emulsion. Initially, the aqueous phase, consisting of distilled water, glycerin, and preservatives (Nipagin and Nipasol), and the oil phase, containing Polawax (emulsifying wax), cetyl alcohol, and liquid paraffin, were heated separately to 75 °C (±5 °C). The aqueous phase was gradually introduced into the oil phase under mechanical stirring at 600 rpm, continuing until emulsification was achieved. The mixture was then allowed to cool under continuous stirring to 25 °C (±5 °C), ensuring proper phase inversion and stability of the emulsion.

At 40 °C, the propolis hydroethanolic extract of straw bees (Scaptotrigona aff. postica) was incorporated into the base at a concentration of 10% *w*/*v* during the cooling phase to maintain the bioactivity of the propolis compounds. The formulation was stirred until a homogeneous mixture was obtained. This method ensured the proper dispersion of propolis in the ointment, resulting in a stable product. The inclusion of Nipagin (methylparaben) and Nipasol (propylparaben) as preservatives enhanced the shelf-life of the formulation, maintaining its physicochemical stability and preventing microbial growth.

### 4.6. Animals

The study was approved by the CEUA (Ethics Committee on the Use of Animals) of the Federal University of Pará (Protocol nº 4539300720). Male Wistar rats (160–200 g, 8–20 weeks old) were housed under controlled conditions (25 ± 2 °C, 12 h light/dark cycle) with ad libitum access to food and water. Seven animals were separated for each group, with each animal in the respective group presenting 3 superficial lesions on the back. During treatment, the animals were kept in individual boxes. The cream was applied every day for 7 days until euthanasia.

#### 4.6.1. Experimental Procedures of Induced Wounds

The procedure was performed as follows: the animals were anesthetized intraperitoneally with ketamine hydrochloride and xylazine hydrochloride, at doses of 100 and 5 mg/kg, respectively. For sedation, a highly volatile inhalable compound, isoflurane, was used. After anesthetic induction, epilation was performed in the dorsal region, followed by asepsis of the area with 2% iodinated alcohol. Lesions were prepared using a 0.6 cm diameter “punch,” excising a fragment of skin in the caudo-cranial direction. Sterile gauze was used to control hemostasis.

**Figure 6:**
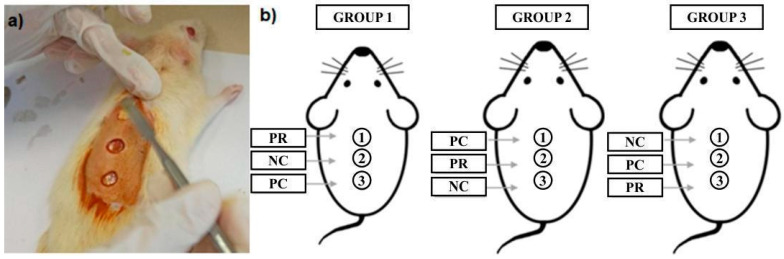
(**a**) Topic administration of samples in wounds induced in the dorsal region of the rat; (**b**) schematic Illustration of cream applications for each sample group: PR (propolis-based cream); NC (neutral cream as negative control); PC (collagenase cream as positive control).

These injuries were subjected to the following treatments: negative control (NC): neutral cream was used without the active ingredient; positive control (PC): Kollagenase^®^ ointment (collagenase 0.6 U/g with chloramphenicol 0.01 g/g), a commercial medicine commonly used for wound healing, was applied; test group (PR): cream containing 10% propolis extract concentration was used. This concentration was considered the most effective based on previous studies in Figure 6 [32,33].

Three lesions were induced in each animal to compare the degree of healing within the same animal, as comparisons between lesions might differ due to variations in surrounding blood circulation and recovery time.

#### 4.6.2. Macroscopic Evaluation

The ImageJ software was used to determine the wound’s diameter and macroscopic aspects, with diameter in mm^2^. Data were categorized and entered into spreadsheets for later comparison between numerical groups (G1, G2 and G3) and sample groups. Data were observed over all seven days of the experiment. After euthanizing, the animals were photographed at about 35 cm from the surgical area. The images were inserted into the software mentioned above to measure the diameter of each lesion.

### 4.7. Microscopic Evaluation

#### 4.7.1. Sample Collection

After the euthanasia of the animals in a bell saturated with ethyl ether vapors, a segment of skin approximately 0.5 cm long by 1.0 cm wide was removed from the irradiated areas and injured sites.

#### 4.7.2. Embedding in Paraffin

After storage for 48 h of collected materials, the paraffin embedding technique was performed. The technique required adequate immersion of the wounds and their respective fluids in different concentrations of alcohol, with each sample conditioned for 40 min in each concentration of alcohol, consisting of seven phases for removing the solvent: insertion of the material for dehydration in 70% alcohol, then 75%, 80%, 85%, 90%, 95%, and 100%; this step aims to carry out the complete dehydration of the sample. It is worth emphasizing that the samples cannot be inserted directly into 100% alcohol; it must be performed gradually, as described above; otherwise, there will be deformation and alteration of the internal positions of the organelles (portions of cell elements).

After the dehydration step, the samples were placed in Xylol and embedded by paraffin incorporation. In short, excised tissue was made with the aid of a scalpel blade in the wrapping of the wound samples, and then the sample was immersed in the mold of molten paraffin in an oven at an average temperature of 70 °C. After 30 min, the clean paraffin was poured and melted into the formed mold and the piece was inserted inside the paraffin. After the paraffin solidifies, the sample is incorporated into cold water, leaving it for about 20 min so it does not form crystals that cause complications regarding its homogeneity. From this stage, the blocks were taken for microtomy and consequent obtaining of the sections, which were then collected on glass slides. In the case of paraffin inclusion, after microtomy, the tissue is treated with xylol again to remove the paraffin and rehydrated, so that it can be submitted to staining by hematoxylin/eosin (HE) methods.

### 4.8. Histopathology

Considering that the tissue sections were colorless after microtomy, the staining step aims to contrast the tissue structures. After staining, the slides were mounted; that is, the fragments were protected by covering them with a glass coverslip. This is incorporated into the slide through sealing reagents such as Entellan. After this step, the slides could be observed microscopically.

## 5. Conclusions

This experimental study presented the efficacy of the propolis-based cream derived from a native stingless bee reared in açai monoculture in induced wound healing over 7 days. A therapeutic action was observed with faster recovery of reticular fibers and collagen type I and III in the induced wound of propolis-treated rats, possibly due to the bioactive compounds present in the propolis. To date, no propolis-based cream reared in açai monoculture has been described so far.

## Figures and Tables

**Table 1:** Content of total phenols and total flavonoids. Results expressed as equivalents of gallic acid and quercetin, respectively, in relation to the hydroethanolic extract of propolis (*m*/*v*).

Assay	Propolis Hydroethanolic Sample (%)	Coefficient of Variation (%)	Requirement (MAPA)
^1^ Total phenol content	5.04%	0.60	Min. 0.25%
^2^ Flavonoid content	1.91%	0.15	Min. 0.50%

^1^ Expressed as gallic acid equivalent to propolis extract (*m*/*m*). ^2^ Expressed as quercetin equivalent to propolis extract (*m*/*m*); MAPA (Brazilian Ministry of Agriculture, Livestock, and Supply).

**Table 2:** Gravimetric analysis expressed by crude propolis content. Percentage calculated from the values expressed in grams, on the mass of raw propolis (*m*/*m*).

Assay	Average	Coefficient of Variation (%)	Requirement (MAPA)
Loss due to desiccation	7.8%	0.10	Max. 8%
Ash content	3.19%	0.15	Max. 5%

**Table 3:** Compounds identified from GC-MS analysis of the propolis ethanol extract of *Scaptotrigona aff. postica* reared in açaí monoculture.

Peak #	RT (min)	Area (%)	Name of Compound	Molecular Formula	MW (g/mol)	Ref.
1	38.307	0.28	1-heptacosanol	C_27_H_56_O	396	
2	38.728	0.32	heneicosane	C_21_H_44_	296	
3	41.369	0.59	1-heptacosanol	C_27_H_56_O	396	
4	45.081	0.30	thunbergol	C_20_H_34_O	290	
5	48.808	0.29	lanosta-8,24-dien-3-one	C_30_H_48_O	424	
6	49.201	0.66	lanosterol	C_30_H_50_O	426	
7	49.293	0.28	ursa-9(11),12-dien-3-one	C_30_H_46_O	422	
8	49.850	13.16	4,4,6a,6b,8a,11,11,14b-octamethyl-1,4,4a,5,6,6a,6b,7,8,8a,9,10	C_30_H_48_O	424	[16]
9	49.980	0.31	stigmasta-5,24(28)-dien-3-ol, (3.beta.,24Z)-	C_29_H_48_O	412	
10	50.086	1.01	6.beta.Bicyclo [4.3.0]nonane, 5.beta.-iodomethyl-1.beta.-isoprop	C_15_H_25_I	332	
11	50.264	7.09	beta.-Amyrin	C_30_H_50_O	426	[17]
12	50.609	32.64	lup-20(29)-en-3-one	C_30_H_48_O	424	[18]
13	51.039	17.21	lupeol	C_30_H_50_O	426	[19]
14	51.391	1.37	9,19-cyclolanostan-3-ol, 24-methylene-, (3.beta.)-	C_31_H_52_O	440	
15	51.586	2.54	olean-12-en-3-ol, acetate, (3.beta.)-	C_32_H_52_O_2_	468	[20]
16	51.703	1.53	9,19-cyclolanostan-3-ol, 24-methylene-, (3.beta.)-	C_32_H_52_O	440	
17	51.805	0.74	lup-20(29)-en-3-one	C_30_H_48_O	424	
18	52.367	9.93	lup-20(29)-en-3-ol, acetate, (3.beta.)-	C_32_H_52_O	468	[21]
19	53.224	0.85	humulane-1,6-dien-3-ol	C_15_H_26_O	222	
20	54.075	0.63	9,19-cyclolanost-25-en-3-ol, 24-methyl-, (3.beta.,24S)-	C_31_H_52_O	440	
21	54.278	1.20	acetic acid, 4,4,6a,6b,8a,11,12,14b-octamethyl-14-oxo-1,2,3,4,4	C_32_H_50_O_3_	482	
22	55.153	1.20	uvaol	C_30_H_50_O_2_	442	
23	56.763	0.49	9,19-cyclolanost-23-ene-3,25-diol, (3.beta.,23E)-	C_30_H_50_O_2_	442	
24	62.292	2.06	olean-12-en-3-ol, acetate, (3.beta.)-	C_32_H_52_O_2_	468	[22]
25	63.937	1.07	lup-20(29)-en-3-ol, acetate, (3.beta.)-	C_32_H_52_O_2_	468	
26	68.741	0.69	octacosyl acetate	C_30_H_60_O_2_	452	
27	72.250	1.59	olean-12-en-3-ol, acetate, (3.beta.)-			

## Data Availability

Data Availability are available from the authors.
